# What Is the Color of Milk and Dairy Products and How Is It Measured?

**DOI:** 10.3390/foods9111629

**Published:** 2020-11-08

**Authors:** Bojana Milovanovic, Ilija Djekic, Jelena Miocinovic, Vesna Djordjevic, Jose M. Lorenzo, Francisco J. Barba, Daniel Mörlein, Igor Tomasevic

**Affiliations:** 1 Faculty of Agriculture, University of Belgrade, Nemanjina 6, 11080 Belgrade, Serbia; idjekic@agrif.bg.ac.rs (I.D.); jmiocin@agrif.bg.ac.rs (J.M.); tbigor@agrif.bg.ac.rs (I.T.); 2 Institute of Meat Hygiene and Technology, Kaćanskog 13, 11000 Belgrade, Serbia; vesna.djordjevic@inmes.rs; 3 Centro Tecnológico de la Carne de Galicia, Parque Tecnológico de Galicia, 32900 San Cibrao das Viñas, Ourense, Spain; jmlorenzo@ceteca.net; 4 Preventive Medicine and Public Health, Food Sciences, Toxicology and Forensic Medicine Department, Faculty of Pharmacy, University of Valencia, 46100 Burjassot, Valencia, Spain; francisco.barba@uv.es; 5 Department of Animal Sciences, University of Göttingen, D-37075 Göttingen, Germany; daniel.moerlein@uni-goettingen.de

**Keywords:** milk color measurement, colorimeter, computer vision system, illuminant, aperture

## Abstract

Exactly six-hundred (600) scientific articles that report milk and milk products’ color results in scientific journals in the last couple of decades were reviewed. Thereof, the greatest part of the articles derived from Europe (36.3%) and Asia (29.5%). The greatest share of researchers used Minolta colorimeters (58.8%), while 26.3% of them used Hunter devices. Most reports were on cheese (31.0%) followed by fermented products (21.2%). Moreover, the highest number of papers reported color data of milk and milk products made from cow’s milk (44.81%). As expected, goat’s cheese was the brightest (L* = 87.1), while cow’s cheese was the yellowest (b* = 17.4). Most importantly, it appeared that color research results reported were often impossible to replicate or to interpret properly because of incomplete description of the methodology. In some of the manuscripts reviewed, illuminant source (61.0%), aperture size (93.8%), observer angle, and number of readings (over 70% of all cases) were not reported. It is therefore critical to set rules regarding the description of the methodology for (milk) color research articles in order to ensure replicability and/or comparison of studies.

## 1. Introduction

Appearance attributes such as color, size, shape, and visual texture often contribute to consumers’ buying decisions [1]. For many food product categories, color conveys information to the consumer about a product’s sensory properties (e.g., taste or flavor) or else about prime brand attributes such as premium, natural, or healthy [2]. For example, the color of cheese is, amongst other factors, related to its age. Typically, a longer maturation is associated with both increased levels of volatile compounds that make up flavor as well as with darker appearance. Thus, the lightness of cheese represents a visible cue, which affects purchase intent depending on individual preferences. This is illustrated by the findings of Jo et al. [3], who comprehensively assessed, amongst other sensory and physico-chemical traits, the color of various young to aged Gouda cheeses thus varying in color lightness (L* of 85 to 67) and related their properties with consumer acceptance. As a result, U.S. consumers preferred Gouda cheeses with a light color; the appearance of some cheeses was clearly disliked according to Just About Right (JAR) ratings—some cheeses were clearly rated as too light or too dark, which illustrates the importance of understanding the impact of color on consumer perception/liking [3]. Hence, color is an important trait of cheese even though it was found that the pleasantness of mouth feel and flavor outweighs the importance of appearance with respect to overall liking [4].

The direction of preferences depends on the type of dairy product. For example, contrary to the cheese example above, increased lightness was found to be detrimental to consumer acceptance for the case of goat milk yogurts in Brazil [5].

Furthermore, for other dairy products such as milk–fruit beverages, sour cream, vanilla ice cream, milk and soymilk vanilla beverages, and butter the relevance of color and appearance for consumer acceptability was demonstrated [6,7,8,9]. This is especially noteworthy as the color variability (L*, a*, b*) was rather low in the case of sour cream.

The color of the milk product can be indicator of physico-chemical changes. In this context, color changes particularly during storage time are vital quality measures and, thus, instrumental color analysis has been widely used in identifying color variations in dairy products over the storage [10].

Furthermore, color of the foods must be analyzed with utmost accuracy. Due to the subjective perception of color and its variety from observer to observer, color measuring devices are mostly used in order to have a reliable and objective color determination [11].

Instrumentally, color measurement can be carried out using conventional instruments and computer vision systems. Commonly used colorimeters are Minolta Chromameter and Hunter Lab colorimeter. There are three attributes for color description namely: hue, lightness, and chromaticity. Hue (h) is a color parameter in which colors are defined as red, green, blue, and yellow. Lightness (L*) is a parameter for color brightness by which we can perceive light and dark color. Chromaticity (C*) or colorfulness represents the color sensation; when color is fully saturated, the color is considered to be in its purest version. Each color can be represented by numerous color spaces. A color space is a model that can be used to characterize as many colors as our vision system can possibly distinguish. Typically, there are many types of color spaces, but instrumental spaces are used for color devices, such as the colorimeters and spectrophotometers [12]. Many of these spaces are standardized by the Commision International d’Eclaraige (CIE) under a series of standard conditions. Instrumental spaces are CIELab, and CIELu*v*, and CIEXYZ spaces. Hunter Lab and CIELAB L*a*b* are two frequently used color spaces for the analysis of colorimeter information, where L* defines lightness or darkness, a* redness or greenness, and b* yellowness or b* blueness. The main difference between these two-color systems is that the Hunter (L, a, b) equations use the square root of CIE X-Y-Z values for L, a, b calculation, whereas the CIE (L*, a*, and b*) equations use the cubic root [13]. Moreover, researchers can report Hunter data from a Minolta instrument, both instruments can convert data into difference color spaces—XYZ, Yxy, L*a*b*, Hunter Lab, and so on. There are various numbers of factors influencing on milk color parameters such as illuminant, observer, aperture size, instrument type, and so on. However, only a few researchers report all the procedures used for color determination. 

In this paper, we had the intention of analyzing reported factors that affect instrumental color data gathering. Ideally for data and research to be meaningful to other researchers, a common method would be developed to measure the color of milk and its products. In order to have a method by which results can be compared between different articles, the following parameters need to be included in an article, the type of illuminant, aperture size, observer angle, type of instrument, number of readings per sample, and calibration procedure.

## 2. Materials and Methods

### 2.1. Inclusion Criteria

We included studies investigating the scientific articles written in English that evaluated the color of dairy products published in the last decade from 2008 to 2019.

### 2.2. Information Source

Papers were retrieved from various databases (Web of Science, ScienceDirect, Scopus, Google Scholar, SpringerLink, Mendeley, Wiley online library, PubMed).

### 2.3. Search Strategy

The color data collection was achieved using the following keywords:

1. Milk color evaluation
2. Hunter Lab milk color values
3. CIE L*a*b* dairy color values
4. Instrumental milk color measurement
5. Instrumental dairy products/cheese color assessment
6. Physicochemical characteristics of milk and milk products

**Figure 1:**
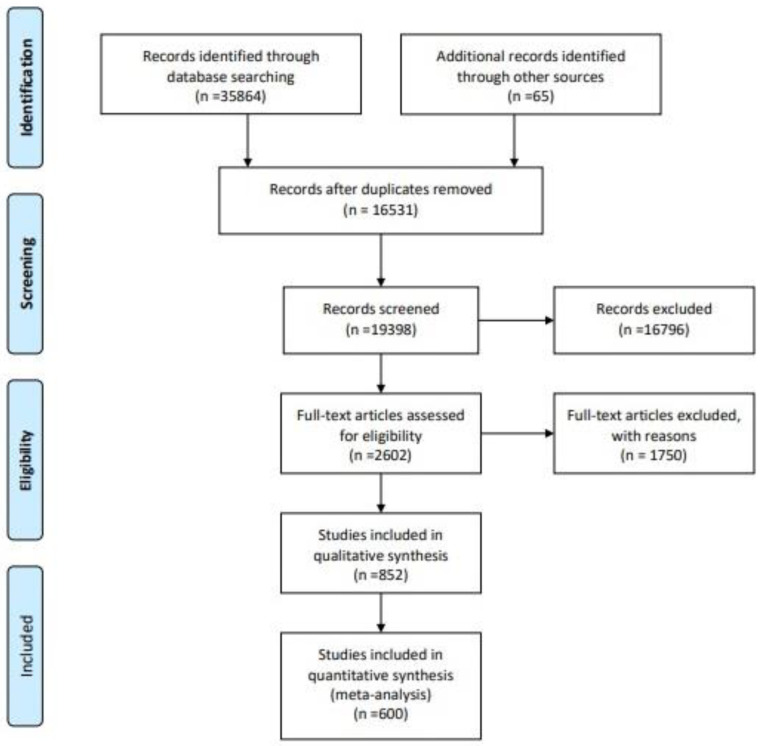
Search strategy and article selection process according to the Preferred Reporting Items for Systematic Reviews and Meta-Analyses (PRISMA) guidelines [14].

Further studies were examined through manual searches of reference lists of selected studies and previously published papers (Figure 1).

### 2.4. Data Extraction and Analysis

The following variables were extracted from each color measurement study: the country of origin of the first author’s affiliation, the type of journal, the type of milk product studied, the type of instrument used as well as methodological aspects such as calibration method, type of illuminant (light source), aperture (port size), observation angle, number of technical replicates per sample, and systems of evaluating color parameters. The countries of study were categorized into six continents: Africa, Asia, Europe, North America, South America, and Oceania (including New Zealand and Australia). For further analyses, milk and milk products were categorized in the following manner:

1. Cheese and analogs;
2. Dairy-based desserts (e.g., ice cream, dulce de leche, peda, etc.);
3. Fats and oils (including butter, butter oil, anhydrous milkfat, ghee);
4. Fermented milk products (including flavored yoghurt);
5. Fluid milk and milk products (include all plain and flavored fluid milks based on skim, part-skim, low-fat, and whole milk);
6. Milk and cream powder and powder analogs;
7. Other products (colostrum, infant formula, cream, milk gel);
8. Whey and whey products.

Milk and milk products are classified according to the Codex Alimentarius [15] with a modification including other products such as colostrum, infant formula, milk gel.

Data were collected using Microsoft Excel. For descriptive statistical analysis, the frequency and cross-tabulation procedure of SPSS software was used to gather information regarding country by light source, port size by device, and milk and milk products by light source interactions.

## 3. Results and Discussion

### 3.1. Search Strategy

The initial search strategy of the databases (Figure 1) [14] resulted in 35,864 articles with 65 articles identified through other sources, making a total 35,929 manuscripts taken into consideration. Following removal of the same articles appearing in different databases (duplicates) (*n* = 16,531) we were left with 19,398 manuscripts that were screened. After applying excluding criteria and removing the articles missing the data about color (*n* = 18,546), 852 manuscripts were subjected to full-text analysis. Additionally, 252 manuscripts were excluded after reading the full text as they were considered irrelevant, mainly because although they contained information about the color of milk and dairy products, it was only descriptive and without exact tristimulus values reported. At the end of the selection process, 600 papers were selected for quantitative analysis. A flow diagram of the selection procedure is included in Figure 1 [14].

### 3.2. Type of Products Studied and Origin of Publication

Our observations showed that the highest number of articles derived from Europe, followed by Asia, North America, South America, Africa, and Oceania (Table 1). Among the papers examined, the majority of studies (88 articles) were derived from the Journal of Dairy Science and 84 articles were from the International Journal of Dairy Technology, whereas 64 were acquired from International Dairy Journal and 59 from LWT (Table 1). Furthermore, there are many journals marked as “other” (the percentage of each article is less than 2%), including American Journal of Animal and Veterinary Sciences, Animal, Australian Journal of Dairy Technology, Dyes and Pigments, Food and Nutrition Sciences, Food Bioscience, Food Packaging and Shelf Life, Food Quality and Preference, and so on (Table 1).

Cheeses (186 articles) were the most commonly analyzed milk product followed by fermented milk products, fluid milk, milk and cream powder, and their analogs, respectively. Other milk products in milk color analysis included colostrum, infant formula, cheesecake, milk gel, and milk microcapsules (Table 2).

### 3.3. Instrument Type Used for Color Determination of Milk and Milk Products

In our research, Minolta colorimeters (Chiyoda-ku, Tokyo, Japan) were used most often (353 articles) to access the color of milk and milk products. Hunter Associates Laboratory (Reston, VA, USA) equipment was used in 158 articles, whereas 25 articles published evaluating milk color with an alternative method such as computer vision systems (Table 3). In our literature analysis, there were other types of devices including Gardner, Data Color, Colorgard system, MOM, LUCI™, Macbeth, etc. with 56 articles. A small number (8 articles) of articles have not reported the brand of instrument utilized to measure milk color. Our investigation is in concurrence with the conclusion of Pathare et al. [13] that Minolta colorimeters are widely used for food color measurements over the Hunter and other types of instruments.

A large number of color measurement differences in literature data were explained by variation of settings and operating conditions even when the same instruments or methodology was used. Cheng et al. [16] demonstrated relevant differences in the lightness or darkness (L*), the red- or greenness (a*), and the yellow- or blueness (b*) values when comparing a Hunter vs. (CIE) color system on the analysis of milk-based beverages. When comparing the Hunter vs. CIE system, it was found that the Hunter instrument was more sensitive to L*, whereas the CIE system was more sensitive to the variations of b* [17].

### 3.4. Calibration Procedure

Instrument calibration and re-calibration are crucial for reliable and accurate data collection. There are different calibration techniques, which may vary by model and brand. Typically, calibration is based on scans of standardized black and white plates. Thereby, a quantitative relationship is established between values indicated by the measurement device and the corresponding values represented by the reference material [18].

Calibration has to be performed before each measurement analysis and should be reported in the manuscripts. However, our results showed that 60.5% of articles did not report about calibration procedures used.

### 3.5. Light Source (Illuminant) Used for Instrumental Color Determination of Milk Products

The light source (also known as illuminant) as well as the angle of observation of the object studies greatly affect the perceived color. The illuminant represents the color temperature of a light source. The illuminants recommended for the measurement of food products by the CIE are illuminant A (2848 K; tungsten-filament lighting), B (4900 K; direct sunlight), C (6800 K; average daylight from the total sky), and D65 (6500 K; spectral distribution of mid-day sun). Accordingly, illuminants D50, D55, and D75 indicate a color temperature of 5000, 5500, and 7500 K, respectively.

When the illuminant was reported and only in 39% of the manuscripts we have reviewed (Table 4), D65 was utilized most frequently (177 out of 235 articles), which corresponds to the findings of Kortei and Akonor [19]. Illuminants A, C, and others such as compact fluorescent lamp (CFL) or fluorescent cool-white (FCW) were noticed in 12, 14, and 32 articles, respectively. It is important to note, however, that the highest number (366) of articles did not report the type of illuminant. Although D65 is the reference illuminant spectrum most used for calculating CIELAB values [20], we would like to stress that it represents a light bluish colored light source (it accentuates blue and subdues green and red) that is not “ideal” for color measurements of milk and dairy products. We believe that the reason for the predominant use (75%) of illuminant D65, in 177 of 235 articles that have it reported (Table 4), is that D65 and C are the only illuminant options for Minolta colorimeters, which were used in the greatest number of articles 58.8% (Table 3). The other reasons is that D65 is used in color matching applications of paints, plastics, textiles, inks, automotive, and other manufactured products and is commonly used as a primary light source in color measurement instrumentation such as Minolta that was not designed for food color evaluation, originally. The preferred illumination used for milk and dairy product color evaluation is still to be investigated and recommended. So far, we can only be sure that it cannot be illuminant A because it accents on the proportion of red wavelengths, and it is therefore recommended for evaluating meat color [21].

### 3.6. Aperture Size of the Instrumental Device for Color Determination

Port size, otherwise known as the aperture size, can influence the results significantly. Using various port sizes resulted in different color data due to the differences in reflectance measurements [22]. Selecting an appropriate aperture size is inherently associated with the size of the sample being evaluated. Aperture sizes can range from 8 mm to more than 3.18 cm. The selection of the largest aperture size is recommended that allows multiple measurements (at least three are recommended) of the same sample with a uniform color. If samples have a non-uniform appearance, selection of the aperture size that covers only color uniform parts of the sample is advisable [23]. For instance, the larger aperture size results in fewer variations in the measurements or restricts the measurements that can be taken. When computer vision system is used for color evaluation, the size of the sample is defined by the software capabilities used for the purpose of extracting tristimulus values from digital images. For example, in case Adobe Photoshop is used, then the color sampler tool is used in order to create persistent pixel value readouts that are displayed in the info panel and can display up to four color sample point readouts in an image. With the color sampler tool, one can monitor pixel color values at fixed points in an image using one of the options: Point Sample, 3 × 3 Average, 5 × 5 Average, 11 × 11 Average, 31 × 31 Average, 51 × 51 Average, or 101 × 101 Average. The Point Sample option samples a single pixel color value only. Since digital images of the food samples are taken in RAW format (minimally processed data from the image sensor of a digital camera) with the resolution of 300 pixels per inch, we can easily calculate that one pixel is equal to 0.0085 cm. Therefore, the maximum sample size (101 × 101 pixels) that can be measured in one technical replicate is equivalent to 0.85 × 0.85 cm.

Since a range of port sizes has been used to evaluate milk color (Table 5), the same sample could have resulted in different color values due to the differences in reflectance measurements [24]. In the present review, the majority of articles examined (563 articles) did not report information on the aperture size. When this information was included, a port size of 8 mm (18 articles) was most commonly used (Table 5).

### 3.7. Observation Angle

Some instruments provide various observation angles. The standard observer is a mathematical representation of the average color vision of the human population. Most common are 2° and 10° observers. However, little is known on the effect of observation angle on the results of milk color assessment. A large proportion of articles (440 articles) did not report the angle of observation (Table 6). However, when it was reported, the dominant angle was 10° (123 articles). The second most used angle was 2° (24 articles).

### 3.8. Technical Replicates (Number of Readings)

The number of readings is necessary for repeating and reproducing measurements because the increase of technical replicates improves the precision of color assessment [25]. In the articles surveyed herein, we found the following number of readings: at least 2 readings per sample (9 articles), 3 readings (44 articles), 4–6 readings (80 articles), 8 readings (6 articles), 10–12 readings (23 articles), and more than 12 readings (3 articles). Most studies (435 articles) did, however, not report the number of readings (Table 7).

The prescribed minimum number of technical replicates for all instrumental color measuring devices is three [26], although this number may increase depending on the devices used, as in the case of HunterLab MiniScan, where it is suggested to be four [27], or with Nix Pro Color Sensor device, where it is advocated to be a minimum of 7 replicates [28]. To the best of our knowledge, this matter was not explored in regard to computer vision color measuring systems, and therefore, we would advise the use of at least three replicates as with the case of other instrumental color measuring devices.

### 3.9. Data for Color Coordinates Tristimulus Methods

In our review, most of the articles (292 articles) reported color data using the CIE L*a*b* color system to calculate lightness, redness, and yellowness, whereas the Hunter Lab color scale was used in 94 articles. In food color measurement, researchers frequently use the L*a*b* system if they are looking at the “true” human eye perception of color [11].

Many articles also calculated other color parameters such as hue angle (70 articles), chroma (82 articles), yellowness (10 articles), browning index (10 articles), whiteness (24 articles), and total color difference (102 articles).

The tone symbol, h*, has the sexagesimal degree (°), and it is defined according to the following mathematical function. Hue angle (h°) refers to the degree of the dominant spectral component, such as red, green, and blue, and ranges from 0° to 360°. An angle of 0° or 360° represents red Hue, while angles 90°, 180°, and 270° represent yellow, green, and blue Hue, respectively. Combining a* and b*, h° better represents the color; it is calculated based on formula (1) [29]:

(1) $$•\;\text{°}\mathrm{Hue}\;=\;\tan^{-1}{\;(a}^{*}{/b}^{*})$$

The chroma (C*) represents the vividness or saturation of a color [29] and is defined as follows: (2):

(2) $$•\;C^{*}{\;=\;(a}^{*2}{\;+\;b}^{*2}{)}^{0.5}$$

The whiteness of milk products is often the most critical color characteristic. The whiteness index (WI) indicates the degree of whiteness and mathematically combines lightness and yellow–blue into a single term. It is widely measured using the following equation according to Vargas et al. [30]:

(3) $$•\;\mathrm{WI}\;=\;100\;-\;{\left({\left({100-L}^{*}\right)}^{2}{\;+\;a}^{*}{}^{2}{\;+\;b}^{*}{}^{2}\right)}^{0.5}$$

In addition to the previously mentioned color models, spectral reflectance measurements (400 to 700 nm) are also a proper measure of whiteness in milk [31]. Some articles reported reflectance values (obtained by spectrophotometers) at wavelengths (360–750 nm) at 5, 10, or 20 nm resolution with 18 articles. Likewise, a few articles noted using X-Y-Z (2 articles) or L-c-h coordinates (4 articles).

The browning index (BI) is defined as brown color purity and is one of the most common indicators of browning in food products containing sugar [32]. The BI is calculated using the following expression [33]:

(4) • BI = 100 × (x - 0.31/0.17)

(5) $$•\;\mathrm{Where}\;X\;=\;\frac{\left(a*\;+\;1.75L\right)a}{(5.645L\;+\;a*\;-\;3.012b*)}$$

The yellowing index (YI) is used as a color measurement related to browning index.

(6) $$•\;\mathrm{YI}\;=\;142.86{\;\times \;b}^{*}/L^{*}$$

Color changes can be measured as total color difference (ΔE). Total color difference indicates the magnitude of color difference between any two samples using the following equation according to Fernandez-Avila et al. [34]:

(7) $$•\;{\Delta E}^{*}\;=\;\left[{\left(\Delta L^{*}\right)}^{2}\;+\;{\left(\Delta a^{*}\right)}^{2}\;+\;{\left(\Delta b^{*}\right)}^{2}\right]{\;}^{1/2}$$

Two colors can be distinguished by the human eye depending on their total color difference: (ΔE < 1) color differences that could not be perceptible to the human eye, (1 < ΔE <3) minor color differences that could be perceptible to the human eye, and (ΔE < 3) color differences that could be perceptible to the human eye [35].

### 3.10. Milk Color Data

The comparative observation of color was achieved by determining the average of the chromatic components (L*a*b*) that were represented by tables in the papers. We investigated control samples to determine whether differences exist among milk products according to the animal species for each milk group, with the exception of “other” and dairy-based desserts due to the variety and the complex composition, which makes the comparison difficult. 

Regarding milks, the color of goat’s milk was closer to that of deer’s than that of other species, both being brighter (higher L* coordinates) than camel’s, mare’s, sheep’s, and cow’s (Table 8). As for the redness, the highest a* values had a milk mixture (a* = 3.1 ± 8.7) resulting in more “red” appearance, while the lowest a* values had deer’s milk (a* = −3.0 ± 0.0) indicating more green color. In terms of yellowness (b* coordinate), the highest value had a mixture (b* = 11.3 ± 9.1), followed by deer’s milk (b* = 8.4 ± 0.0), sheep’s (b* = 7.5 ± 1.3), cow’s (b* = 7.5 ± 4.4), goat’s (b* = 5.5 ± 0.0), camel’s (b* = −0.2 ± 0.0), and mare’s milk (b* = −2.3 ± 2.4). The standard variations may be due to the possible chemical alterations in the milk (the carotenoid, protein, and riboflavin content). Furthermore, it has been reported that goat’s milk is brighter because of its ability to convert β-carotene to vitamin A [36]. The “a*” and “b*” parameters are affected by factors associated with milk’s natural pigment amount. For instance, lutein and zeaxanthin are present in high quantity in green herbage [37] and these can be assimilated into sheep’s milk causing yellowish color [38]. However, milk carotenoids are responsible for the yellow color of cow’s milks in comparison to sheep’s and goat’s milks, which are devoid of β-carotene. The probable reason for differences in color data can be the fact that milk color is affected by many variables such as genetic and nongenetic factors. In the case of the milk color, it is possible that differences are linked to the diet [39] and breed [40], parity and month of test [41], and seasonal calving [42]. For example, Jersey breeds had brighter color than others (Holsteins, Friesians, Norwegian Reds, and Montbéliarde cows), while the milk of Friesians, Jerseys, Norwegian Reds, and Montbéliardes was greener than that of Holsteins. Moreover, Jersey cows had yellower milk than Holsteins. Otherwise, the milk of Montbéliarde cows had bluer color than the milk of Holsteins. This could be described by the ability of the cow to transform carotene into vitamin A and the higher fat amount present in Jersey milk relative to that in Friesian, Holstein, Montbéliarde, and Norwegian Red cows [41]. However, this trend was opposite to the results reported by Solah et al. [31] regarding Holstein–Friesian cows in Western Australia. Obvious differences among parities, lactation time, and month of test also exist in milk color [42] in the cooler period, resulting in higher milk color intensity. Feed composition also can be a possible factor for color variations in milk and dairy products. The observed seasonal variation of milk fat color could be explained by the deviations in concentration of β-carotene concerning changing pasture composition. Moreover, the color of milk fat is important because it affects the color of dairy products including butter and cheese. The presence some infection can be related to the milk color traits as suggested by Viguier et al. [43], who reported more reddish color of bovine milk in the case of clinical mastitis. Technological processes, as well as conditions and time of storage, can result in color changes in dairy products. Homogenization causes the higher values in lightness (cream, milk), whereas thermal processes may cause either an increase or a decrease in parameter L*. An increase in whiteness arose from denaturation of β-lactoglobulin and its conjugation to j-casein. Popov-Raljić et al. [44] examined color changes in Ultra High Temperature (UHT) milk with a fat content of 3.2% stored at a temperature of 20 ± 5 °C for 90 days. They noted changes in lightness from 89.88 to 77.15, in redness from –3.26 to 2.12, and in yellowness from 9.27 to 7.06. Additionally, storage environment and technological procedure can also change the physical structure of milk resulting in color variations [45], especially in the case of L* values [46].

When it comes to cheeses, the brightest was goat’s cheese (L* = 87.1 ± 14.8) following by mixture (L* = 85.9 ± 5.5), buffalo’s (L* = 85.2 ± 11.5), sheep’s (L* = 83.3 ± 6.4) and cow’s cheese (L* = 82.6 ± 9.2) (Table 9). Redness and yellowness exhibited the highest values for cow’s cheese (a* = 0.3 ± 9.1 and b* = 17.4 ± 10.0). Goat’s cheese is generally whiter than cow’s cheese [47]. The observed standard deviations may be due to the chemical composition (smaller fat globule and a total conversion of β-carotene into vitamin A). Furthermore, buffalo’s cheese had the highest negative a* values (with the exception of mixture) due to its chromaticity portion of green color, caused by the presence of the blue–green pigment (biliverdin), which is present in buffalo’s milk and absent in cow’s milk [48]. Regarding the yellow parameter, it is higher in cheeses from cows than in those from sheep or goats. This investigation is in concurrence with the studies reported by Raynal-Ljutovac et al. [49]. In cheeses made from the milk of other animal species, color is influenced by treatments, and the variables that affect this trait the most are treatment heat, pressure grade, and holding period [50]. Some researchers related increases in L* and b* of the cheese surface to microstructural changes. Koca et al. [51] stated that pressure-treated higher-pressure levels and longer pressure-holding times resulted in significantly lower a* values and higher b* values, making treated Turkish brined white cheese more greenish and yellowish. On the other hand, the decrease in cheese whiteness during ripening was associated with the concentration of cheese components. Furthermore, proteolysis that occurs during ripening can transform casein into a more soluble state and can cause a decrease in whiteness [52]. A similar trend was noticed in previous studies, a whiter color of milk after sonication treatment (higher L* value) was observed due to the homogenization and reduction of fat globules to smaller sizes and the further association with casein micelles [53,54]. The increase in the a* and b* color parameters is mainly due to the increase of the concentration of the cheese components owing to the dehydration throughout the ripening process [55], and a similar trend was found for lightness (L*) during the first two months of ripening [56].

Observing butter color data (Table 9), butter made from goat’s milk was lighter in color (L* = 95.0 ± 0.0) than butter made from cow’s milk (L* = 84.9 ± 9.9). Regarding a* values, goat’s and cow’s butter were equal (a* = −1.4 ± 3.1 and a* = −1.4 ± 0.0, respectively), while cow’s butter was more yellowish (b* = 24.3 ± 7.5). It is well-known fact that the natural yellow color of butter is primarily because of the carotene (lycopene), vitamin A, and other pigments [57], and therefore, the huge standard deviations of color values of the butter can be because of the carotene content in the feed. Moreover, previous studies reported that butter made from cow’s milk had a higher yellow color [57]. Process-related factors such as ripening and storage are relevant in the color of butter. The butter color is usually changed from yellow to light yellow [58]. Owing to the lack of carotenoids in goat’s milk, this trend was not observed in the goat’s cream butters. A study of Kristensen et al. [59] reported that a very high storage temperature resulted in a darker butter color. Differences in butter color result from variation in the color of butter fat, variation in the size of fat globules, the presence or absence of salt, the condition of working the butter [60], the type of packaging, and storage temperature. The color of dairy products also depends on the type and amount of coloring added.

Concerning fermented products, the L* values were in the range 66.9 ± 2.2 (goat’s kefir) to 93.0 ± 0.0 (buffalo’s kefir) (Table 9). In contrast, in terms of redness, values were between −3.8 ± 1.7 (fermented milk made from cow’s milk) and 6.0 ± 6.6 (fruit yoghurt made from cow’s milk), and yellowness values ranged from 0.3 ± 7.2 (fruit yoghurt made from cow’s milk) to 11.2 ± 7.5 (set-type yoghurt made from cow’s milk). Variations in color readings are in agreement with the different degree of gel opacity. Therefore, it can be related to the casein proportion and its aggregation level [25]. Moreover, Erkaya et al. [61] investigated yoghurts produced using milk from different species and noticed greater total solids, protein, and lipid content in sheep’s milk yoghurt when compared to cow’s and goat’s milk yoghurts. In addition, the a* parameter of ewe’s milk is lower compared with that of bovine milk [62], due to the fact that plasma carotenoids are lower in ewe’s milk than in bovine milk [38]. The higher the protein amount of sheep’s milk, the greener will be the yogurt. Additionally, one can said that color values of fermented milks were more affected by the type of milk (milk substitution) rather than by the used starter cultures. Mani-Lopez et al. [63] stated that the color parameters L*, a*, and b* of yogurts and fermented milks remained almost constant during storage. The opposite trend was observed by Hilali et al. [64], i.e., that parameter L* increased over time, indicating that the yogurt became lighter, possibly due to the increase in milk fat content, which is responsible for the white color. The redness parameter tended to become more green and yellowness became less yellow with the advance of the milking time, possibly due to the presence of dried plants in the range and other influencing factors on the concentration of xanthophylls in range species, such as exposure to sunlight [38].

In relation to the color of milk powder (Table 9) the lightness, redness, and yellowness values of cow’s, goat’s, sheep’s, and the mixture milk powder were 88.6 ± 10.1, 0.2 ± 6.8, 11.8 ± 6.1; 89.2 ± 6.0, −1.6 ± 0.7, 11.0 ± 2.8; 87.9 ± 3.6, −3.6 ± 0.4, 8.8 ± 6.5; and 78.7 ± 21.3, 1.1 ± 6.5, 13.0 ± 17.0, respectively. The L*a*b* values differed among powders not only because of different source of milk and their differences in chemical composition but also due to compositional differences in the retentates/concentrates and variations in the industrial process such as type of dryer, number of drying stages, and drying conditions employed during their manufacture [65]. High-protein powders have grayish–white color because of the yellowish–white color of skimmed milk powder. Maillard reactions lead to changes in food color. For example, lower L* values could be the result of the formation of brown pigments in the sugar protein mixture and darker sample [66]. Temperature can also be a potential reason for different results in color. For example, the unheated skim milk concentrate was whitish with an L* value (brightness of the sample) of 79.3 ± 2.2. The redness was negative for all samples, which indicates a shift to a slight greenish color. The Maillard products in the later stage not only lead to the brown color of the milk product but are also seen as an initiator for cross-linking of proteins in powdered milk products [67].

The color coordinates (L*a*b*) of whey products were 83.0 ± 15.1, 1.2 ± 6.2, and 7.9 ± 4.7, respectively. In general, powders come in a variety of colors depending on the ingredients used. In addition, changes in color coordinates can be attributed to the different opacity level of gels. The greater the opacity, the higher the sample brightness and the less pure the color [68]. The color values observed in this observation may be related to the fact that whey is a greenish–yellow by-product [69] and color is also affected by Maillard reaction during drying processes [70].

### 3.11. Interaction between Country and Instrument

**Figure 2:**
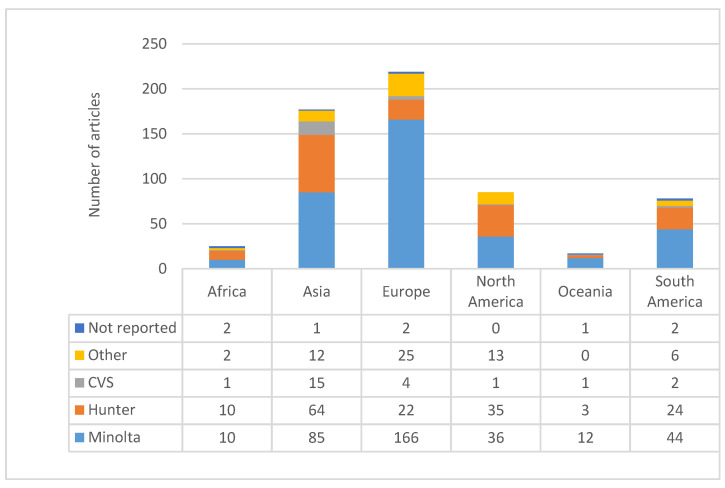
Interaction of countries with type of device for milk color assessment.

The Minolta devices were the most frequently used instruments in 58.8% of all articles examined, and this colorimeter was the most dominant for measuring milk color within majority of countries with the exception of Africa (Figure 2).

In articles from Europe, Oceania, and South America, the Minolta instrument was reported in 75.8%, 70.6%, and 56.4% of articles, respectively. European authors were much less likely to use “other” (11.4%), Hunter (10.0%), or computer vision systems (CVS, 1.8%). Oceanian authors used the Hunter (17.6%) instruments more often than CVS (5.9%). In South America, Minolta was the most frequent device (56.4%) in comparison to the Hunter (30.8%), “other” (7.7%), and CVS (2.6%). Authors from Africa used Minolta similarly often (40.0%) and Hunter (40.0%) over the “other” (8.0%) and the one reported CVS (4.2%). African, European, and South American authors had the same article number of non-reported instruments (25.0% of articles not reporting devices for each continent). Finally, Asia and Oceania had non-reported instruments at 12.5% for each continent. There are various factors that can influence the instrument a researcher chooses, including the cost of the device, brand, its availability, and capabilities needed (settings), etc. Furthermore, the choice of which instrument to use will depend on the food material and type of application. Ideally, the instrument of choice meets the goals of the study, considering ethical, budgetary, and time constraints, among others, though the price varies by model, for example, spectrophotometers typically cost more than a colorimeter. Moreover, there are many other influencing factors to consider, such as reliability and validity that represent fundamental features in the evaluation of any measurement instrument or tool for a good research [71].

## 4. Conclusions

According to color data observed in this review, we found that the majority of papers investigated milk and milk products obtained from cow’s milk. Color measurements are frequently reported based on different color units even for the same dairy product, making it difficult to trace and compare research data between different investigations. There is still currently a lack of international standards for milk color measurement. Even more important, findings from this review indicated that a majority of the articles failed to include relevant information necessary to replicate and accurately interpret instrumental color results. Therefore, when reporting studies on instrumental milk color evaluation, as a minimum, the following information should be included: instrument used, calibration, illuminant, aperture size, observation angle, and number of readings taken per sample (technical replicates). We strongly believe that the results of this review may serve as a capstone for food authority controls or policy-makers and food chemists to develop a reliable and internationally accepted protocol of color measurement of milk and dairy products for quality control.

## Figures and Tables

**Table 1:** Data for journals and countries studied for measuring milk color.

Journals	Number of Articles (%)
Other ^1^	203 (33.8%)
Journal of Dairy Science	88 (14.7%)
International Journal of Dairy Technology	84 (14.0%)
International Dairy Journal	64 (10.7%)
LWT-Food Science and Technology	59 (9.8%)
Food Research International	22 (3.7%)
Food Chemistry	21 (3.5%)
Innovative Food Science and Emerging	20 (3.3%)
Food Hydrocolloids	14 (2.3%)
Dairy Science and Technology	13 (2.2%)
Journal of Food Engineering	12 (2.0%)
**Country of research**	
Europe	218 (36.3%)
Asia	177 (29.5%)
North America	84 (14.0%)
South America	79 (13.2%)
Africa	25 (4.2%)
Oceania	17 (2.8%)

^1^ Other included all journals with less than 2% of the articles.

**Table 2:** Data for milk categories.

Milk and Milk Products	Number of Articles (%)
Cheese	186 (31.0%)
Fermented products	127 (21.2%)
Fluid milk and milk products	102 (17.0%)
Milk and cream powder	49 (8.2%)
Dairy-based desserts	48 (8.0%)
Whey and whey powder	47 (7.8%)
Other ^1^	32 (5.3%)
Fats and oils (e.g., butter)	16 (2.7%)

^1^ Other milk products studied included colostrum, infant formula, cheesecake, cream, milk gel, and milk microcapsules.

**Table 3:** Data for color devices.

System Used for Color Determination	Number of Articles (%)
Minolta	353 (58.8%)
Hunter	158 (26.3%)
Other ^1^	56 (9.0%)
CVS (Computer vision system)	25 (4.8%)
Not reported	8 (1.3%)

^1^ Other means devices studied included Gardner, Data Color, Colorgard system, MOM, LUCI™, Macbeth.

**Table 4:** Data for illuminant studied for measuring milk color.

Illuminant	Number of Articles (%)
Not reported	366 (61.0%)
D65	177 (29.5%)
Other ^1^	32 (5.3%)
C	14 (2.3%)
A	12 (2.0%)

^1^ Means other illuminants studied included compact fluorescent lamp (CFL) or fluorescent cool-white (FCW). C: 6800 K; average daylight from the total sky; A: 2848 K; tungsten-filament lighting.

**Table 5:** Data for port size studied for measuring milk color.

Aperture Size	Number of Articles (%)
Not reported	563 (93.8%)
8 mm	18 (3.0%)
45 mm	5 (0.8%)
More than 50 mm	5 (0.8%)
10–25 mm	4 (0.7%)
30–35 mm	2 (0.3%)
50 mm	2 (0.3%)
4 mm or less	1 (0.2%)

**Table 6:** Data for observer studied for measuring milk color.

Observer	Number of Articles (%)
Not reported	440 (73.3%)
10	123 (20.5%)
2	24 (4.0%)
0	6 (1.0%)
More than 45	4 (0.7%)
3–8	2 (0.3%)
45	1 (0.2%)

**Table 7:** Data for technical replicates studied for measuring milk color.

Number of Readings	Number of Articles (%)
Not reported	435 (72.5%)
4–6	80 (13.3%)
3	44 (7.3%)
10–12	23 (3.8%)
2	8 (1.3%)
7–8	7 (1.2%)
15–20	2 (0.3%)
More than 20	1 (0.2%)

**Table 8:** Milk color data (mean ± st. dev).

Source of Milk		Cow’s (*n* = 41)	Goat’s (*n* = 1)	Sheep’s (*n* = 3)	Camel’s (*n* = 1)	Deer’s (*n* = 1)	Mare’s (*n* = 2)	Mixture (*n* = 23)
**Milks** **(*n* = 72)**	L*	81.0 ± 8.1	86.0 ± 0.0	79.9 ± 8.9	67.8 ± 0.0	89.2 ± 0.0	73.5 ± 14.3	64.8 ± 22.3
	a*	−1.5 ± 3.0	−2.1 ± 0.0	−2.4 ± 1.3	−2.0 ± 0.0	−3.0 ± 0.0	−2.2 ± 0.2	3.1 ± 8.7
	b*	7.5 ± 4.4	5.5 ± 0.0	7.5 ± 1.3	−0.2 ± 0.0	8.4 ± 0.0	−2.3 ± 2.4	11.3 ± 9.1

**Table 9:** Color data of milk products (mean ± st.dev).

Source of Milk		Cow’s (*n* = 93)	Goat’s (*n* = 20)	Sheep’s (*n* = 19)	Buffalo’s (*n* = 5)	Mixture (*n* = 4)
Cheeses (*n* = 141)	L*	82.6 ± 9.2	87.1 ± 14.8	83.3 ± 6.4	85.2 ± 11.5	85.9 ± 5.5
	a*	0.3 ± 9.1	0.0 ± 2.8	−0.7 ± 2.8	−3.0 ± 3.2	−4.3 ± 3.6
	b*	17.4 ± 10.0	8.2 ± 4.4	15.4 ± 3.8	12.3 ± 7.3	14.5 ± 4.4
		(*n* = 6)	(*n* = 1)			(*n* = 5)
Butter (*n* = 12)	L*	84.9 ± 9.9	95.0 ± 0.0	-	-	40.3 ± 39.2
	a*	−1.4 ± 3.1	−1.4 ± 0.0	-	-	−1.5 ± 2.2
	b*	24.3 ± 7.5	4.9 ± 0.0	-	-	9.7 ± 9.0
		(*n* = 34)	(*n* = 8)	(*n* = 4)	(*n* = 1)	(*n* = 15)
Yoghurt (*n* = 62)	L*	86.2 ± 11.0	86.6 ± 6.0	84.0 ± 17.7	84.8 ± 0.0	64.6 ± 27.9
	a*	−1.1 ± 4.9	−0.8 ± 2.2	−18.8 ± 28.3	−1.1 ± 0.0	−0.0 ± 6.6
	b*	9.0 ± 5.3	12.1 ± 7.0	11.8 ± 7.9	8.1 ± 0.0	9.7 ± 8.5
		(*n* = 9)				(*n* = 4)
Fermented milks (*n* = 13)	L*	70.5 ± 18.0	-	-	-	76.7 ± 28.2
	a*	−3.8 ± 1.7	-	-	-	2.8 ± 3.0
	b*	8.9 ± 5.0	-	-	-	9.3 ± 10.4
		(*n* = 8)				
Set-type yoghurt (*n* = 8)	L*	86.6 ± 8.5	-	-	-	-
	a*	−1.8 ± 4.4	-	-	-	-
	b*	11.2 ± 7.5	-	-	-	-
		(*n* = 1)	(*n* = 2)		(*n* = 1)	
Kefir (*n* = 4)	L*	91.6 ± 0.0	66.9 ± 2.2	-	93.0 ± 0.0	-
	a*	−2.2 ± 0.0	−1.7 ± 0.1	-	−1.7 ± 0.0	-
	b*	6.2 ± 0.0	5.1 ± 0.2	-	6.5 ± 0.0	-
		(*n* = 4)				
Fruit yoghurt (*n* = 4)	L*	69.8 ± 13.7	-	-	-	-
	a*	6.0 ± 6.6	-	-	-	-
	b*	0.3 ± 7.2	-	-	-	-
		(*n* = 17)	(*n* = 3)		(*n* = 2)	(*n* = 13)
Milk powder products (*n* = 35)	L*	88.6 ± 10.1	89.2 ± 6.0	-	87.9 ± 3.6	78.7 ± 21.3
	a*	0.2 ± 6.8	−1.62 ± 0.7	-	−3.56 ± 0.4	1.13 ± 6.5
	b*	11.8 ± 6.1	11.0 ± 2.8	-	8.8 ± 6.5	13.0 ± 17.0
		(*n* = 20)				(*n* = 6)
Whey products (*n* = 26)	L*	83.0 ± 15.1	-	-	-	67.5 ± 25.1
	a*	1.2 ± 6.2	-	-	-	2.4 ± 9.5
	b*	7.9 ± 4.7	-	-	-	6.3 ± 6.4

- No data reported.

## References

[1] Conti-Silva A.C., Souza-Borges P.K. (2019). Sensory characteristics, brand and probiotic claim on the overall liking of commercial probiotic fermented milks: Which one is more relevant?. Food Res. Int..

[2] Spence C., Velasco C. (2018). On the multiple effects of packaging color on consumer behaviour and product experience in the ‘food and beverage’ and ‘home and personal care’ categories. Food Qual. Prefer..

[3] Jo Y., Benoist D.M., Ameerally A., Drake M.A. (2018). Sensory and chemical properties of Gouda cheese. J. Dairy Sci..

[4] Ritvanen T., Lampolahti S., Lilleberg L., Tupasela T., Isoniemi M., Appelbye U., Lyytikäinen T., Eerola S., Uusi-Rauva E. (2005). Sensory evaluation, chemical composition and consumer acceptance of full fat and reduced fat cheeses in the Finnish market. Food Qual. Prefer..

[5] Costa M.P., Monteiro M.L.G., Frasao B.S., Silva V.L.M., Rodrigues B.L., Chiappini C.C.J., Conte-Junior C.A. (2017). Consumer perception, health information, and instrumental parameters of cupuassu (Theobroma grandiflorum) goat milk yogurts. J. Dairy Sci..

[6] Krause A.J., Lopetcharat K., Drake M.A. (2007). Identification of the characteristics that drive consumer liking of butter. J. Dairy Sci..

[7] Cadena R.S., Cruz A.G., Faria J.A.F., Bolini H.M.A. (2012). Reduced fat and sugar vanilla ice creams: Sensory profiling and external preference mapping. J. Dairy Sci..

[8] Shepard L., Miracle R.E., Leksrisompong P., Drake M.A. (2013). Relating sensory and chemical properties of sour cream to consumer acceptance. J. Dairy Sci..

[9] Fernández-Vázquez R., Stinco C.M., Hernanz Vila D., Heredia F.J., Chaya C., Vicario I.M. (2017). Internal preference mapping of milk–fruit beverages: Influence of color and appearance on its acceptability. Food Sci. Nutr..

[10] Ryan J., Hutchings S.C., Fang Z., Bandara N., Gamlath S., Ajlouni S., Ranadheera C.S. (2020). Microbial, physico-chemical and sensory characteristics of mango juice-enriched probiotic dairy drinks. Int. J. Dairy Technol..

[11] Leon K., Mery D., Pedreschi F., Leon J. (2006). Color measurement in L*a*b* units from RGB digital images. Food Res. Int..

[12] Rossel R.A.V., Minasny B., Roudier P., Mcbratney A.B. (2006). Color space models for soil science. Geoderma.

[13] Pathare P.B., Opara U.L., Al-Said F.A.J. (2012). Color measurement and analysis in fresh and processed foods: A review. Food Bioproc. Technol..

[14] Moher D., Liberati A., Tetzlaff J., Altman D.G. (2009). Preferred reporting items for systematic reviews and meta-analyses: The PRISMA statement. PLoS Med..

[15] Codex Alimentarius. General Standard for Food Additives. http://www.fao.org/gsfaonline/docs/CXS_192e.pdf.

[16] Cheng N., Barbano D.M., Drake M.A. (2018). Hunter versus CIE color measurement systems for analysis of milk-based beverages. J. Dairy Sci..

[17] Whetzel N. Measuring Color Using Hunter L, a, b versus CIE 1976 L* a* b*. https://support.hunterlab.com/hc/enus/articles/204137825-Measuring-Color-using-Hunter-Lab-versus-CIE-1976-Lab-AN-1005b.

[18] Phillips S.D., Estler W.T., Doiron T., Eberhardt K.R., Levenson M.S. (2001). A careful consideration of the calibration concept. J. Res. Natl. Inst. Stand. Technol..

[19] Kortei N., Akonor P. (2015). Correlation between hue-angle and color lightness of gamma irradiated mushrooms. Ann. Food Sci. Technol..

[20] MacDougall D.B., Gulrajani M.L. (2010). Colour measurement of food: Principles and practice. Colour Measurement Principles, Advances and Industrial Applications.

[21] Tapp W., Yancey J., Apple J. (2011). How is the instrumental color of meat measured?. Meat Sci..

[22] MacDougall D.B., Pearson A.M., Dutson T.R. (1994). Color of meat. Quality Attributes and Their Measurement in Meat, Poultry and Fish Products.

[23] AMSA Meat Color Measurement Guidelines. https://meatscience.org/publications-resources/printed-publications/amsa-meat-color-measurement-guidelines.

[24] Yancey J.W.S., Kropf D.H. (2008). Instrumental reflectance values of fresh pork are dependent on aperture size. Meat Sci..

[25] Mason R.L., Gunst R.F., Hess J.L. (2003). Statistical Principles in Experiment Design Statistical Design and Analysis of Experiments; with Application to Engineering and Science.

[26] Honikel K.O. (1998). Reference methods for the assessment of physical characteristics of meat. Meat Sci..

[27] (2012). Measuring Meat Steaks, Fillets and Patties Using the MiniScan EZ 45/0 LAV. www.hunterlab.com.

[28] Holman B., Collins D., Kilgannon A., Hopkins D. (2017). The effect of technical replicate (repeats) on Nix Pro Color Sensor™ measurement precision for meat: A case-study on aged beef colour stability. Meat Sci..

[29] Bermúdez-Aguirre D., Mawson R., Versteeg K., Barbosa-Cánovas G.V. (2009). Composition properties, physicochemical characteristics and shelf life of whole milk after thermal and thermo-sonication treatments. J. Food Qual..

[30] Vargas M., Chafer M., Albors A., Chiralt A., Gonzalez-Martinez M. (2008). Physicochemical and sensory characteristics of yoghurt produced from mixtures of cows’ and goats’ milk. Int. J. Dairy Technol..

[31] Solah V.A., Staines V., Honda S., Limley H.A. (2007). Measurement of milk color and composition: Effect of dietary intervention on Western Australian Holstein-Friesian cow’s milk quality. J. Food Sci..

[32] Buera M., Lozano R., Petriella C. (1986). Definition of color in the non-enzymatic browning process. Die Farbe.

[33] Erbay Z., Koca N. (2015). Effects of whey or maltodextrin addition during production on physical quality of white cheese powder during storage. J. Dairy Sci..

[34] Fernandez-Avila C., Gutierrez-Merida C., Trujillo A.J. (2017). Physicochemical and sensory characteristics of a UHT milk-based product enriched with conjugated linoleic acid emulsified by Ultra-High-Pressure Homogenization. Innov. Food Sci. Emerg. Technol..

[35] Quintanilla P., Beltran M.C., Molina A., Escriche I., Molina M.P. (2019). Characteristics of ripened Tronchon cheese from raw goat milk containing legally admissible amounts of antibiotics. J. Dairy Sci..

[36] Lucas A., Rock E., Agabriel C., Chilliard Y., Coulon J.B. (2008). Relationships between animal species (cow versus goat) and some nutritional constituents in raw milk farmhouse cheeses. Small Rumin. Res..

[37] Prache S., Cornu A., Berdagué J.L., Priolo A. (2005). Traceability of animal feeding diet in the meat and milk of small ruminants. Small Rumin. Res..

[38] Nozière P., Graulet B., Lucas A., Martin B., Grolier P., Doreau M. (2006). Carotenoids for ruminants: From forages to dairy products. Anim. Feed Sci. Technol..

[39] Langman L. (2009). Calidad Organolética en Leche Expresada en Su Color y Perfil de Olor. Relación de Estos Parámetros con la Incorporación de Antioxidantes Naturales en la Dieta Implementada en las Vacas. Ph.D. Thesis.

[40] Berry S.D., Davis S.R., Beattie E.M., Thomas N.L., Burrett A.K., Ward H.E., Stanfield A.M., Biswas M., Ankersmit-Udy A.E., Oxley P.E. (2009). Mutation in bovine β-carotene oxygenase 2 affects milk color. Genetics.

[41] Scarso S., McParland S., Visentin G., Berry D.P., McDermott A., De Marchi M. (2017). Genetic and nongenetic factors associated with milk color in dairy cows. J. Dairy Sci..

[42] Walker G.P., Wijesundera C., Dunshea F.R., Doyle P.T. (2013). Seasonal and stage of lactation effects on milk fat composition in northern Victoria. Anim. Prod. Sci..

[43] Viguier C., Arora S., Gilmartin N., Welbeck K., O’Kennedy R. (2009). Mastitis detection: Current trends and future perspectives. Trends Biotechnol..

[44] Popov-Raljić J.V., Lakić N.S., Laličić-Petronijević J.G., Barać M.B., Sikimić V.M. (2008). Color changes of UHT milk during storage. Sensors.

[45] Hassan A., Amjad I., Mahmood S. (2009). Microbiological and physicochemical analysis of different UHT milks available in market. Afr. J. Food Sci..

[46] Grigioni G., Biolatto A., Langman L., Descalzo A., Irurueta M., Paez R., Taverna M., Rui M.S. (2010). Color and pigments in milk and dairy. Practical Food Research.

[47] Park Y.W., Park Y.W., Haenlein G.F.W. (2006). Goat milk-chemistry and nutrition. Handbook of Milk of Non-Bovine Mammals.

[48] Abd El-Salam M.H., El-Shibiny S. (2011). A comprehensive review on the composition and properties of buffalo milk. Dairy Sci. Technol..

[49] Raynal-Ljutovac K., Lagriffoul G., Paccard P., Guillet I., Chilliard Y. (2008). Composition of goat and sheep milk products: An update. Small Rumin. Res..

[50] Martínez-Rodríguez Y., Acosta-Muñiz C., Olivas G.I., Guerrero-Beltrán J., Rodrigo-Aliaga D., Sepúlveda D.R. (2012). High hydrostatic pressure processing of cheese. Compr. Rev. Food Sci. Food Saf..

[51] Koca N., Balasubramaniam V.M., Harper W. (2011). High-Pressure Effects on the Microstructure, Texture, and Color of White-Brined Cheese. J. Food Sci..

[52] Chudy S., Bilska A., Kowalski R., Teichert J. (2020). Color of milk and milk products in CIE L*a*b* space. Medycyna Weterynaryjna.

[53] Bermúdez-Aguirre D., Barbosa-Cánovas G.V. (2008). Study of butter fat content in milk on the inactivation of Listeria innocua ATCC 51742 by thermo-sonication. Innov. Food Sci. Emerg. Technol..

[54] Bermúdez-Aguirre D., Corradini M.G., Mawson R., Barbosa-Cánovas G.V. (2009). Modeling the inactivation of Listeria innocua in raw whole milk treated under thermo-sonication. Innov. Food Sci. Emerg. Technol.

[55] Ávila M., Garde S., Nuñez M. (2008). The influence of some manufacturing and ripening parameters on the color of ewes’ milk cheese. Milchwissenschaft.

[56] Diezhandino I., Fernández D., Sacristán N., Combarros-Fuertes P., Prieto B., Fresno J.M. (2016). Rheological, textural, color and sensory characteristics of a Spanish blue cheese (Valdeón cheese). LWT.

[57] Queirós M.S., Grimaldi R., Gigante M.L. (2016). Addition of olein from milk fat positively affects the firmness of butter. Food Res. Int..

[58] Kaya A. (2000). Properties and stability of butter oil obtained from milk and yoghurt. Food Nahrung.

[59] Kristensen D., Boesen M., Jakobsen U.L., Månsson L., Erichsen L., Skibsted L.H. (2000). Oxidative and color stability of salted sour cream dairy spread compared to salted sweet cream dairy spread. Milchwissenschaft.

[60] Hettinga D. (2005). Bailey’s Industrial Oil and Fat Products.

[61] Erkaya T., Şengül M.A. (2012). Comparative Study on Some Quality Properties and Mineral Contents of Yoghurts Produced from Different Type of Milks. Kafkas Universitesi Veteriner Fakultesi Dergisi.

[62] Mazloomi S.M., Shekarforoush S.S., Ebrahimnejad H., Sajedianfard J. (2011). Effect of adding inulin on microbial and physic-chemical properties of low fat probiotic yogurt. Iran. J. Vet. Res..

[63] Mani-López E., Palou A., López-Malo (2014). Probiotic viability and storage stability of yogurts and fermented milks prepared with several mixtures of lactic acid bacteria. J. Dairy Sci..

[64] Hilali M., Iñiguez L., Knaus W., Schreiner M., Wurzinger M., Mayer H.K. (2011). Dietary supplementation with nonconventional feeds from the Middle East: Assessing the effects on physicochemical and organoleptic properties of Awassi sheep milk and yogurt. J. Dairy Sci..

[65] Meena G.S., Singh A.K., Arora S., Borad S., Sharma R., Gupta V.K. (2017). Physico-chemical, functional and rheological properties of milk protein concentrate 60 as affected by disodium phosphate addition, diafiltration and homogenization. J. Food Sci. Technol..

[66] Morales F.J., van Boekel M.A. (1998). A study on advanced maillard reaction in heated casein/sugar solutions: Color formation. Int. Dairy J..

[67] Le T.T., Holland J.W., Bhandari B., Alewood P.F., Deeth H.C. (2013). Direct evidence for the role of Maillard reaction products in protein cross-linking in milk powder during storage. Int. Dairy J..

[68] González-Martınez C., Becerra M., Cháfer M., Albors A., Carot J.M., Chiralt A. (2002). Influence of substituting milk powder for whey powder on yoghurt quality. Trends Food Sci. Technol..

[69] Carvalho F., Prazeres A.R., Rivas J. (2013). Cheese whey wastewater: Characterization and treatment. Sci. Total Environ..

[70] De Castro-Cislaghi F.P., Carina Dos Reis E.S., Fritzen-Freire C.B., Lorenz J.G., Sant’Anna E.S. (2012). Bifidobacterium Bb-12 microencapsulated by spray drying with whey: Survival under simulated gastrointestinal conditions, tolerance to NaCl, and viability during storage. J. Food Eng..

[71] Mohajan H. (2017). Two Criteria for Good Measurements in Research: Validity and Reliability. Ann. Spiru Haret Univ. Econ. Ser..

